# Transposable elements in the era of data science EMBO course: a call for open, accessible, and collaborative TE resources

**DOI:** 10.1186/s13100-026-00412-0

**Published:** 2026-07-31

**Authors:** Alexandre Rossi Paschoal, Fabiana Rodrigues de Goes, Wojciech Makałowski, Gökhan Karakülah, Rebecca V Berrens, Emmanuelle Lerat, Cristina Vieira, Shujun Ou, Anna-Sophie Fiston-Lavier, Marie-Anne Van Sluys, Romain Guyot

**Affiliations:** 1 Rosalind Franklin Institute, Harwell Science and Innovation Campus, Didcot, OX11 0QS UK; 2 Department of Computer Science (DACOM), Federal University of Technology – Paraná (UTFPR), Cornélio Procópio, Paraná 86300-000 Brazil; 3 Department of Genetics, Institute of Experimental Biology, Adam Mickiewicz University, 61-614 Poznan, Poland; 4 Izmir Biomedicine and Genome Center, İzmir, 35340 Türkiye; 5 Izmir International Biomedicine and Genome Institute, Dokuz Eylül University, İzmir, 35340 Türkiye; 6 Department of Biochemistry, Oxford University, Oxford, OX1 3QU UK; 7 Universite Claude Bernard Lyon 1, LBBE, UMR5558, CNRS, VAS, Villeurbanne, F-69622 France; 8 Department of Molecular Genetics, The Ohio State University, Columbus, OH 43210 USA; 9 ISEM, Univ Montpellier, CNRS, IRD, Montpellier, France; 10 Institut Universitaire de France (IUF), Paris, France; 11 Departamento de Botânica, Instituto de Biociências, Universidade de Sao Paulo, São Paulo, 05508-090 SP Brazil; 12 UMR DIADE, IRD, CIRAD, Université de Montpellier, Montpellier, France

## Abstract

This community letter represents a collective outcome from the EMBO Practical Course “Transposable Elements in the Era of Data Science” held at the Rosalind Franklin Institute from 12 to 16 May 2025, Didcot, United Kingdom (https://meetings.embo.org/event/25-transposable-elements*).* Written by course speakers together with an instructor representative, it reflects key discussions about the urgent need for open, accessible, and community-driven resources for transposable element (TE) research. This letter outlines key areas where collective action is imperative to accelerate our understanding of TEs and their profound impact on biological systems. In particular, the letter highlights limitations of existing databases, particularly Repbase, and calls for a new open-access platform that also supports non-model species and interoperates with large-scale genomic initiatives. It also underscores the crucial discussion on TE curation and the role of artificial intelligence (AI) in advancing TE research and advocates for a collaborative infrastructure to support the future of TE genomics. A central theme emerging from these discussions was the urgent need for enhanced open science practices, particularly concerning data sharing, tool development, and the establishment of robust, accessible community resources. To this end, we propose a set of strategic suggestions accompanied by practical actions to advance TE research. We hope this letter contributes to the community of TE researchers to discuss the latest advancements and persistent challenges in TE research in a sustainable and equitable manner.

## Introduction

The study of transposable elements (TEs) has advanced significantly in recent decades, revealing their central role in genome structure, function, and evolution. As a group of researchers convened at the EMBO course “Transposable Elements in the Era of Data Science” [1], we find ourselves at a turning point. The exponential growth of genome sequencing initiatives, including the Darwin Tree of Life (Sanger Institute; https://www.darwintreeoflife.org*)*, Vertebrate Genomes Project (VGP; https://vertebrategenomesproject.org*)*, Bat1K (https://bat1k.com/*)*, the Genome Atlas of European Biodiversity (ERGA; https://www.erga-biodiversity.eu/ [2]), and the 10,000 Plants initiative (10KP [3]), which are driven in part by the Earth BioGenome Project, is generating unprecedented volumes of genomic data from a wide range of species, particularly non-model organisms. This transformation offers a unique opportunity and an urgent imperative for the TE research community to rethink how we identify, annotate, curate, genotype, and share TE data.

The pioneering RepBase database [4], originally established in 1994 by Jerzy Jurka (Genetic Information Research Institute (GIRI)) [5], served for years as a central resource for TE annotation in eukaryotic species. It was truly community-built, with contributions from researchers from different parts of the globe, followed by the review of experts from the GIRI. However, its current paywall model restricts access to many researchers, including those who contributed sequences to the database and those working in underfunded institutions or the Global South. This situation hampers collaborative science, reduces transparency, and risks perpetuating inequality in access to genomic resources. In this context, Dfam [6], another community-built database, has established itself as an essential open-access resource, providing uncurated and curated TE families, consensus sequences, and Hidden Markov Models for sensitive annotation across species. Nevertheless, despite its significant contributions and open-access philosophy, Dfam has two major limitations. First, taxonomic representation across the tree of life is uneven, with a clear taxonomic bias towards vertebrates and plants, and most invertebrate lineages are substantially underrepresented. This gap reflects historical patterns of community engagement and contribution rather than any architectural constraints of the database itself. Second, while the open user submission model is essential for growth, it has also introduced a significant body of uncurated data, where quantity has outpaced quality control.

The recent announcement of a collaboration between Repbase and Dfam has renewed optimism about the future of TE resources [7]. Nevertheless, it is still too early to determine whether this partnership will create a sustainable, open-access ecosystem or whether future licensing or governance changes could restrict community access again. The community can continuously benefit from this initiative if the longevity of the database can be guaranteed.

There are other existing community initiatives that are going in this direction, but they do not fully address all the resource challenges discussed here. TE Hub (https://tehub.org/*)* [8] provides an important catalog and a collection of educational resources that ease the identification and usage of existing tools, databases, events, and other TE-related topics. While the TransposonsWorldwide Slack brings a community channel for discussion among the TE community. However, discussions hosted on Slack are not fully open or easily archived as a persistent scientific resource. This reflects a broader challenge for the field: establishing sustainable, open, and infrastructure-level solutions that extend beyond community discussion platforms.

We strongly advocate the establishment of an open-access, FAIR-compliant, community-driven resource that develops into a federated, interoperable, community-governed infrastructure integrating existing and emerging initiatives into a unified ecosystem. This resource must prioritize the inclusion of TE annotations and curated data, including non-model species across the tree of life, and support interoperability with genomic consortia. Beyond serving as a sequence repository, it should also function as a centralized database for standardized annotation tools, taxonomic classification, and metadata integration across species, thereby supporting ongoing efforts in comparative genomics and evolutionary biology.

In parallel, the TE field is poised to benefit significantly from the integration of AI. Machine learning approaches can aid in the detection, classification, and functional prediction of TEs across divergent lineages. However, realizing this potential requires robust, curated training datasets and transparent benchmarking practices-yet another reason why an open, collaborative infrastructure is essential. As Barbara McClintock envisioned the genome as a “sensitive organ of the cell that senses the environment,” it is now incumbent on us to ensure that our community builds the tools to explore this vision at scale and across the tree of life. Open science, data sharing, and equitable infrastructure are not only ideals-they are prerequisites for meeting the challenges of contemporary genome biology. We invite the broader genomics and bioinformatics communities to join this effort to help define standards, contribute tools, and share data. Together, we can shape a future where the study of TEs is open, inclusive, and empowered by both human insight and AI. Building on these considerations, we outline a vision for the future of TE research and education:

- Complement and potentially supersede RepBase: By providing a freely accessible alternative that incorporates best practices in curation, annotation, and classification, with universal classification rules applicable across all organisms, facilitating easy comparisons between species, enabling widespread adoption by researchers, and also being responsive to the evolving needs of the community. Proprietary restrictions on key resources hinder collaboration and slow scientific progress.
- Prioritize non-model species: Focus on the systematic annotation and classification of TEs in the rapidly expanding genomic datasets from underrepresented organisms. This is crucial for understanding the diversity and evolutionary dynamics of TEs beyond a few well-studied model systems.
- Integrate with existing and emerging genomic resources: Ensure seamless interoperability with major genome databases and annotation pipelines to facilitate data flow and analysis.
- Foster community-driven TE annotation and curation: Establish a sustainable framework for community-based submission, annotation, and expert curation of TE families through dedicated online platforms. Such a framework should facilitate collaborative review, transparent versioning, and scalable quality control while integrating computational support to streamline expert decision-making. In the long term, advances in AI offer the opportunity to develop next-generation curation systems that assist experts throughout the annotation and validation process.
- Embrace FAIR principles: Design the database to be Findable, Accessible, Interoperable, and Reusable (FAIR), ensuring its long-term utility and impact.
- Apply Artificial Intelligence (AI) in TE Research: AI provides an opportunity (and also a challenge) alongside the TE community to improve the understanding and the structure of these very diverse genomes.
- The Imperative for Open-Source Tools and Data Sharing: The TE research community advocates for open-source tools and data sharing as essential pillars of equitable and reproducible science. As large-scale genomic projects expand, the impact of this data depends on its accessibility. Also, all newly developed tools and resources should remain open and ensure long-term accessibility by depositing materials in independent repositories (e.g., Zenodo or Figshare).
- Education represents a crucial supplementary dimension: We recommend that training initiatives, such as this EMBO course held in Didcot (UK), serve as strategic models to be exported to other locations, ensuring broader accessibility, fostering capacity building, and amplifying long-term impact. Such initiatives could support the development and sharing of key community knowledge and practices, including transposable element classification frameworks, the use of open resources, and the development and application of standardized analysis and quality-control pipelines.

To translate these ideas into practical solutions, we propose the following concrete actions:

- Develop automated TE annotation pipelines: analogous to the NCBI Eukaryotic Genome Annotation Pipeline (EGAP) to enable standardized, reproducible TE annotation for newly generated genome assemblies. These pipelines should be informed by community-established best practices and benchmarking guidelines [9], as promoted by the TE Hub.
- Establish community-based curation infrastructure: including a wiki-style platform of expert volunteers to review, validate, and flag problematic annotations, complemented by standardized submission and quality-control workflows for reference libraries.
- Development of semi-automated and AI-assisted annotation workflows: Expand and integrate semi-automated curation pipelines based on existing tools, such as MCHelper [10] and TEtrimmer [11], to reduce the manual effort required for TE family annotation and validation while improving scalability and reproducibility. These workflows could serve as the foundation for future AI-agent-assisted curation systems, analogous to emerging AI-based approaches developed for genome and gene annotation [12–13].
- Adopt minimum reporting and curation standards for TE annotation studies: Making transparent documentation of annotation strategies, evidence, software versions, reference libraries, and curation procedures a routine component of published work.
- Establish an international TE consortium: The community should coordinate efforts in data submission, expert curation, benchmarking, education, and interoperability among existing resources. TEhub, the TE international initiative, can become the central coordinating hub for these community efforts.
- Reference library: Coordinate reference library development through harmonized submissions to Dfam and other repositories, while establishing community consensus on TE family definitions, evidence criteria, nomenclature, and versioning.
- Benchmarking: Establish a common benchmark dataset, a set of genomes, and evaluation metrics for TE discovery, annotation methods, and curation. A similar idea was discussed in a TE workshop a year ago [14], indicating that it is an old, urgent community request.
- Education: Expand education and training, promoting community-wide adoption of best-practice guidelines, reproducible annotation workflows, and standardized analytical procedures.
- Interoperability: Improve interoperability by adopting common file formats, metadata standards, application programming interfaces (APIs), and reciprocal cross-links among databases, annotation tools, and downstream analysis platforms.
- Exploit advances in genome sequencing technologies: The rapid growth of high-quality genome assemblies generated through long-read sequencing and large-scale initiatives such as the Earth BioGenome Project and the Darwin Tree of Life Project provides an unprecedented opportunity to improve TE discovery, annotation, and comparative analyses across the tree of life.
- Distinguish evidence annotation: have confidence tiers to distinguish manually curated consensus sequences versus uncurated outputs. Dfam would be stronger if every family had a clear label like (i) high-confidence, manually curated; (ii) moderate-confidence, supported by multiple assemblies; (iii) provisional / predicted; and (iv) deprecated / suspicious.
- Broaden species representation: Current TE reference libraries are disproportionately derived from a limited number of well-studied model organisms and clades. To produce more representative reference collections and improve annotation accuracy across biodiversity, future efforts should prioritize the systematic sampling of underrepresented lineages, such as basal metazoans, non-model plants, fungi, reptiles, amphibians, and other phylogenetically and ecologically important taxa.

## Conclusion

The TE research community stands at a pivotal moment, poised to leverage unprecedented genomic data and computational advancements. By committing to open science principles, establishing a new community-driven TE database, and strategically integrating artificial intelligence, we can overcome existing barriers and accelerate our understanding of these fascinating and impactful genomic elements. We urge the scientific community, funding bodies, and publishers to support these initiatives to ensure that the future of TE research is collaborative, equitable, and transformative.

Signed,

Members of the TE community at the EMBO Practical Course 2025 “Transposable Elements in the Era of Data Science” held at the Rosalind Franklin Institute from May 12 to 16, 2025, Didcot, United Kingdom (https://meetings.embo.org/event/25-transposable-elements*).*

## Data Availability

No datasets were generated or analysed during the current study.
